# Comparative Analysis of EO-1 ALI and Hyperion, and Landsat ETM+ Data for Mapping Forest Crown Closure and Leaf Area Index

**DOI:** 10.3390/s8063744

**Published:** 2008-06-06

**Authors:** Ruiliang Pu, Peng Gong, Qian Yu

**Affiliations:** 1 Department of Geography, University of South Florida, 4202 E. Fowler Ave., NES 107, Tampa, FL 33620 USA; Tel.: +1 813 974 1508; Fax: +1 813 974 4808; 2 State Key Lab of Remote Sensing Science, Jointly Sponsored by Institute of Remote Sensing, Applications, Chinese Academy of Sciences, and Beijing Normal University, Beijing China, 100101; Center for Assessment and Monitoring of Forest and Environmental Resources (CAMFER), 137 Mulford Hall, University of California, Berkeley, CA 94720-3114 USA; Tel.: +1 510 642 5170; Fax: +1 510 643 5098; E-mail: gong@nature.berkeley.edu; 3 Department of Geosciences, University of Massachusetts, 611 N Pleasant St., Amherst, MA 01003 USA; Tel.: +1 413 545 2095; Fax: +1 413 545 1200; E-mail: qyu@geo.umass.edu

**Keywords:** Hyperion, ALI, ETM+, Leaf area index, Crown closure, Vegetation index, Texture information, Maximum noise fraction

## Abstract

In this study, a comparative analysis of capabilities of three sensors for mapping forest crown closure (CC) and leaf area index (LAI) was conducted. The three sensors are Hyperspectral Imager (Hyperion) and Advanced Land Imager (ALI) onboard EO-1 satellite and Landsat-7 Enhanced Thematic Mapper Plus (ETM+). A total of 38 mixed coniferous forest CC and 38 LAI measurements were collected at Blodgett Forest Research Station, University of California at Berkeley, USA. The analysis method consists of (1) extracting spectral vegetation indices (VIs), spectral texture information and maximum noise fractions (MNFs), (2) establishing multivariate prediction models, (3) predicting and mapping pixel-based CC and LAI values, and (4) validating the mapped CC and LAI results with field validated photo-interpreted CC and LAI values. The experimental results indicate that the Hyperion data are the most effective for mapping forest CC and LAI (CC mapped accuracy (MA) = 76.0%, LAI MA = 74.7%), followed by ALI data (CC MA = 74.5%, LAI MA = 70.7%), with ETM+ data results being least effective (CC MA = 71.1%, LAI MA = 63.4%). This analysis demonstrates that the Hyperion sensor outperforms the other two sensors: ALI and ETM+. This is because of its high spectral resolution with rich subtle spectral information, of its short-wave infrared data for constructing optimal VIs that are slightly affected by the atmosphere, and of its more available MNFs than the other two sensors to be selected for establishing prediction models. Compared to ETM+ data, ALI data are better for mapping forest CC and LAI due to ALI data with more bands and higher signal-to-noise ratios than those of ETM+ data.

## 1. Introduction

Three revolutionary imagers: Advanced Land Imager (ALI), Atmospheric Corrector (AC) and Hyperspectral Imager (Hyperion), onboard the EO-1 satellite have been collecting multispectral and hyperspectral scenes in coordination with the Enhanced Thematic Mapper Plus (ETM^+^) on Landsat 7 [1]. A significant part of the EO-1 program is to perform data comparisons among Hyperion, ALI and ETM+. Such a comparison is also required by the United States Landsat Data Continuity Mission (LDCM, [2]) to advance the legacy of the Landsat program with the intent of serving science and society. The comparisons are ensured, since the EO-1 orbit matches the Landsat 7 orbit with only one minute delay. Since launching EO-1, such comparisons have been conducted by many researchers who focused on either absolute radiometric values [3-5] or applicabilities of various sensors' data [6-11].

For example, after comparing the retrieved surface reflectances from ALI with those from ETM+ and Landsat-4, 5 TM and considering the fact that ALI is a sensor launched for validation of new sensor technologies, Bryant et al. [5] concluded that the ALI sensor performed extremely well. Chander et al. [3] conducted a cross calibration of ALI and ETM+ sensors and their results of the radiometric comparison indicate that the relative sensor chip assemblies gains agree with the ETM+ visible/near infrared (VNIR) band gains to within 2% and with the short-wave infrared (SWIR) bands to within 4%. In discriminating forests with Hyperion, ALI and ETM+ images, Goodenough et al. [9] compared capabilities of the three sensors' data used for forest classification at various classification levels. Their experimental results indicated that Hyperion (overall accuracy of 90%) outperformed ALI (85%) and ETM+ (75%) in forest classification and that ALI classification results were much better than ETM+. Furthermore, Neuenschwander et al. [6] demonstrated higher classification accuracy of mapping flood features in the Okavango Delta, Botswana when using ALI compared to ETM+. In our previous work to compare the capabilities of the three sensors (Hyperion, ALI and ETM+) by the effect of individual bands on estimating forest crown closure (CC) and leaf area index (LAI), we found the Hyperion data consistently outperformed the ALI and ETM+ data while ALI was better than ETM+ [7].

In this study, we continue the comparative analysis of capabilities of three sensors: ALI, ETM+ and Hyperion by mapping forest CC and LAI at Blodgett Forest Research Station, University of California at Berkeley, in Northern California. LAI quantifies the amount of live green leaf material present in the canopy per unit ground area and is defined as the total one-sided area of all leaves in the canopy within a defined region (m^2^/m^2^) [12] while CC can be defined as percentage of land area covered by the vertical projection of tree crowns. We based on the following two reasons to conduct this further comparative analysis. Firstly, in addition to our previous work [7], we conduct mapping forest CC and LAI and emphasize on spatial analysis of CC and LAI in the study area, rather than focusing on correlation analysis of the three sensors' data with forest CC and LAI. Secondly, instead of using individual bands, various band regions and subsets of bands of the three sensors' data for estimating forest CC and LAI, Vegetation indices (VIs), spectral texture variables (VARs) and maximum noise fractions (MNFs) are extracted from the sensors' data for developing pixel-based models of predicting forest CC and LAI. To do so, mixed coniferous forest CC and LAI measurements were collected at the study area. Data from Hyperion, ALI and ETM+ were used to map the forest CC and LAI. Therefore, the objective for this analysis is to compare capabilities of ALI, ETM+ and Hyperion for mapping forest CC and LAI with selected spectral features and indices extracted from the three sensors' data.

## 2. Study site and sensors' data

### 2.1. Study site

**Figure 1.**
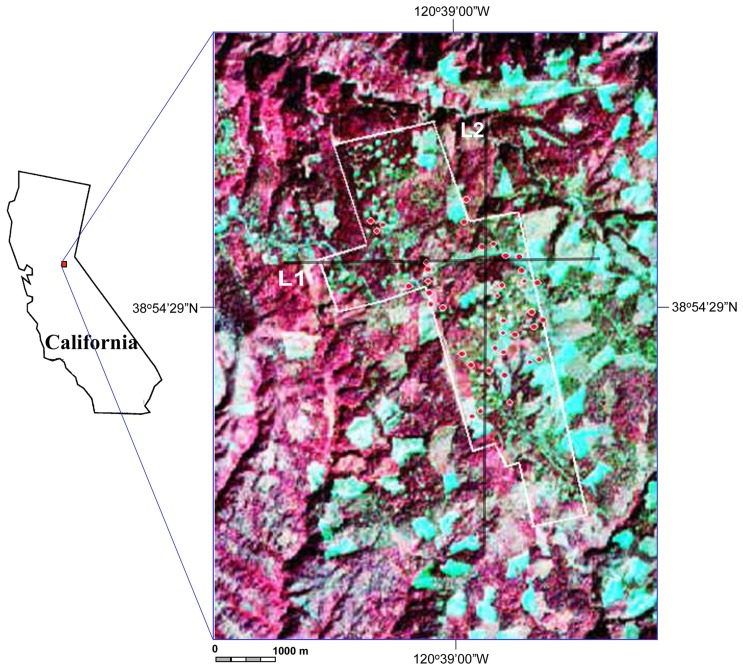
The location of the study site and the positions of plots where forest CC and LAI were measured were marked on the pseudo color composite image of Hyperion (wavelengths 813/681/548 nm vs. R/G/B) in red-fill circle symbols. Label L1 and L2 on the figure present locations of profile analysis for CC and LAI maps (see Figures 3 & 4).

The Blodgett Forest Research Station (120°39′00′W/38°54′29”N) of the University of California, Berkeley, located in the American River watershed on the western slope of the central Sierra Nevada mountain range, El Dorado County, California (Figure 1), was selected as the study site. The Blodgett study area is bounded by a white line in the figure. The vegetation consists of the normal associations of Sierra mixed conifer forest. The major tree species include: Five conifers, Sugar pine (SP, *Pinus lambertiana*), Ponderosa pine (PP, *Pinus ponderosa*), White fir (WF, *Abies concolor*), Douglas fir (DF, *Pseudotsuga menziesii*) and Incense cedar (IC, *Calocedrus decurrens*); and one hardwood, California black oak (*Quercus kelloggii*). A species native to the Sierra Nevada but not found in the Blodgett Forest, Giant sequoia (GS, *Sequoiadendron giganteum*), has been planted at the Blodgett research station since the 1900's. In this study, we measured forest CC and LAI both from mixed coniferous forests with the six conifer species in most compartments at the study site.

### 2.2. Field data collections

We took a total of 38 forest CC and 38 LAI measurements in plots on August 10-11, 2001, two months before the acquisition of the three sensors' data. The measurement plots were typically located within the mixed coniferous forests. The plot size is around 2500-3500 m^2^ to ensure that 2-4 pixels (30 m resolution) are included from each of the three sensors. Two cross lines were laid out with a 50-meter tape in each plot. These lines were aligned along approximately S-N and W-E directions across the plot center. We then measured and summed the intercepted lengths vertically projected by crowns in the overstory. A CC value (%) was calculated through the formula: CC(%) = sum of intercepted crown lengths / total line length. A CC measurement taken from a plot was finally determined by synthetically considering the results measured and visually estimated in forest compartments and interpreted from true-color aerial photographs.

An LAI-2000 Plant Canopy Analyzer (PCA) was used to take LAI measurements at the same plots as for measuring CC values. Each LAI measurement represents an average of ten PCA readings. The locations of PCA readings were selected in each plot based on the canopy closure, age of stands, degree mixed with species and nutrient level so as to make the measurements representative of the variability within the plot. For plots with LAI > 2.0, almost no understory was found while with LAI lower than 2.0 there exists a varying proportion of understory that may have contributed to LAI measurement. Because a sensor always responds to both the understory and overstory within its field- of-view, the LAI measurement in this study has reflected contributions from both understory and overstory. The LAI measurement taken by the PCA is ‘effective’ LAI [12, 13]. Since the effective LAI is less variable and easier to measure than “true” LAI, is an intrinsic attribute of plant canopies [12], and also has a proportional relationship with the “true” LAI [14], we directly use the effective LAI throughout this analysis and refer to it as LAI.

To conduct an atmospheric correction to the ALI and ETM+ data, we also took reflectance measurements in the field from targets of road surface (asphalt and gravel materials), bare soil and young tree canopy (DF, GS, IC, PP, SP and WF) using a FieldSpec®Pro FR (Analytical Spectral Devices, Inc., USA) between 11:30 and 14:30 on August 18, 2002. The instrument consists of three separate spectrometers with spectral range from 350 nm to 2500 nm. All spectra were measured at the nadir direction of the radiometer with a 25° FOV. Depending on the target size, the distance between the instrument's fiber head and its target was 20 cm to 1 m to allow within-target-area radiance measurement. White reference current was measured every 5-10 minutes. Each sample was measured 10 times. To ensure the measurements representing the target, each time we moved the fiber head a little bit but guaranteed the *in situ* measurements taken from the within-target-area.

### 2.3. The characteristics of three sensors and image data acquisition

The detailed descriptions to the characteristics of the two EO-1 sensors: Hyperion and ALI, and ETM+ as well as the EO-1 mission had been provided by Ungar et al. [1] and in Pu et al. [7]. Simple characteristics of the three sensors and number of bands available used for this analysis are summarized in Table 1. The ALI is a ten-band multispectral system with multiple linear arrays embedded in a single sensor chip assembly (SCA) [15]. These bands have been designed to mimic six Landsat bands with three additional bands covering 433-453 nm, 845-895 nm, and 1200-1300 nm (Table 1). The ALI has 30 m resolution for the multispectral pixels and 10 m resolution for the panchromatic pixels. The instrument can represent one 37 km by 100 km land area per image. In this study, we used 9 multispectral bands for comparison with the other two sensors. Hyperion is a high- resolution hyperspectral imager capable of resolving 220 spectral bands (from 0.4 to 2.5 □m) with a 30 m spatial resolution and a nominal spectral resolution of 10 nm. The instrument can represent one 7.5 km by 100 km land area per image and can provide detailed spectral mapping across all 220 bands with high radiometric accuracy. The Hyperion has two spectrometers, one VNIR spectrometer and one SWIR spectrometer. Because of low ratio of signal-to-noise at both spectral ends, heavy water absorption centered around 1400 and 1900 nm and the spectral overlap of the two spectrometers, 75 bands were dropped from original 242. Thus a total of 167 bands (effective bands; were used in this analysis. Operating by a whiskbroom scanning multichannel radiometer, the ETM+ has 6 multispectral VNIR and SWIR bands, one panchromatic band and one thermal band with spatial resolutions of 30 meters for 6 VNIR/SWIR bands, 60 meters for 1 thermal band and 15 meters for 1 panchromatic band. The instrument achieves one 185 km by 185 km land area per image. In this study, we used 6 of these multispectral bands for comparisons purpose.

ALI and Hyperion data covering the study site were acquired on October 09, 2001, around 10:30 a.m. local time. Due to ETM+ data not available on the same day as EO-1 data, the ETM+ data were acquired on October 25, 2001, around 10:30 a.m. local time in this comparative analysis of sensors' data. A set of true color aerial photographs was taken on May 25, 2000 at a nominal scale of 1:8,000, used for validating forest CC and LAI results mapped with the three sensors' data.

## 3. Methods

### 3.1. Retrieving surface reflectance

Atmospheric correction for all the three sensors' data was first conducted to retrieve surface reflectance. With the High Accuracy Atmospheric Correction for Hyperspectral Data (HATCH, *cf.* [16, 17]), atmospheric correction for hyperspectral data of Hyperion was accomplished at the Center for the Study of Earth from Space, Department of Geological Sciences, University of Colorado, USA. The HATCH aims at retrieving surface reflectance spectra of high quality with a reasonable speed. For the ALI and ETM+ data, surface reflectance was retrieved using the Simple Atmospheric Correction method (SAC, *cf.* [18, 19]). In retrieving surface reflectance with SAC, we first needed three at-sensor total radiances simulated with MODTRAN4 [20]. Thereafter, spectral measurements taken from targets in the study area were used to modify the preliminary retrieved surface reflectance. All surface reflectance data retrieved from the three sensors are used in following comparative analysis.

### 3.2. Extraction of spectral features/indices

To develop multivariate regression models with selected spectral features/indices and ground measured CC and LAI data for predicting pixel-based forest CC and LAI, vegetation indices (VIs), spectral texture variables (VARs) and maximum noise fractions (MNFs) were first constructed or extracted from the three sensors' data. In this study, ten VIs were selected and they are NormalizedDifference Vegetation Index (NDVI), Simple Ration Index (SR), ND Water Index (NDWI), Water index (WI), Leaf Chlorophyll Index (LCI), Photochemical Reflectance Index (PRI), Structural Independent Pigment Index (SIPI), Modified Simple Ratio index (MSR), Non-Linear vegetation Index (NLI) and Modified Non-Linear vegetation Index (MNLI). They were constructed using relevant multispectral bands of ALI and ETM+, but for Hyperion, we selected relatively effective bands, most of which are located in NIR and SWIR spectral regions, rather than considering the VIs' original definitions [19]. Three spectral texture variables (defined in variance) were extracted from red and NIR bands of the three sensors and blue band as an additional band for Hyperion. The three bands (only two for ALI and ETM+) used for extracting spectral texture information have a higher correlation with forest CC and LAI than the other bands [7]. The first 20 MNFs, 9 MNFs and 6 MNFs were extracted, respectively, from 167 Hyperion bands, 9 ALI bands and 6 ETM+ bands. The characteristics of the plant canopy are related to the VIs, VARs and MNFs, whose definitions and citation sources have been summarized in Table 2. The reasons of selecting such variables and indices are briefly addressed as follows.

The most commonly used vegetation indices are simple algorithms based on the dissimilar interaction of red and near-infrared (NIR) electromagnetic radiance with vegetation canopies. Among them, the ratio-based normalized difference vegetation index (NDVI, [21]), and the simple ratio vegetation index (SR, [22]) are the most frequently used to correlate with CC, LAI and other canopy structure parameters (e.g., [23-25]). Besides using red and NIR bands, Gong et al. [19] also tested SWIR bands and NIR bands with Hyperion hyperspectral data to construct NDVI and SR VIs and found the VIs constructed with SWIR and NIR bands better than these constructed using red and NIR bands only.

The value of WI increases with leaf water content. This is because the WI compares the leaf (liquid) water absorption band near 970 nm with a reference band at 900 nm, which does not show leaf water absorption [26]. The re-ordered Hyperion bands 47 and 54 are very similar to bands at 970 nm and 900 nm, thus are used to construct the WI. The NDWI is based on leaf (liquid) water absorption band near 1240 nm and a relative nonabsorption reference band near 860 nm [27]. This index also increases with leaf water content. Another index PRI was used as a reliable water-stress index [28]. Given the relationship between canopy water content and canopy CC or LAI, the three water indices should be useful for estimating canopy CC and LAI. The LCI, developed by Datt [29], was found to be a sensitive indicator of chlorophyll content in leaves and was less affected by scattering from the leaf surface and internal structure variation. In consideration of the relationship between canopy CC & LAI and total chlorophyll content and available Hyperion bands for the index, it was also selected as a potential VI. The structure-independent pigment index (SIPI) is correlated with the carotenoid : chlorophyll a ratio [30]. Carotenoids exhibit a well-known absorption peak at 445 nm. Since the three sensors all provide available bands to calculate the index, in considering the carotenoids absorption feature, we hope the index useful in describing the variation of canopy CC and LAI.

Because of possible non-linear relationship between VIs and canopy structure parameters, three nonlinear VIs: MSR [31], NLI [32] and MNLI [19] were considered in the prediction models of CC and LAI. The MSR and MLI non-linear vegetation indices attempt to linearize relationships with surface parameters that tend to be nonlinear. In order to adopt merits from the two VIs to improve their performance correlating with canopy LAI, Gong et al. [19] also tested MNLI by modifying NLI and considering merits of Soil Adjust Vegetation Index coupled with NLI and proved that MNLI had a higher correlation with LAI than either NLI or MSR. While using red and NIR bands for ALI and ETM+ data to construct NLI and MSR, Hyperion bands located in SWIR and NIR (Table 2) were used. The MNLI is only for Hyperion sensor.

Spectral texture is one of the important characteristics used to identify objects (e.g., forest CC and LAI in this analysis) of interest in an image. Unlike spectral features (e.g., VIs), which describe the average tonal variation in the various bands of an image, textural features contain information about the spatial distribution of tonal variations within individual bands [33]. In this practice of mapping CC and LAI, we assumed the spatial distribution of tonal variations of a band image correlated with the spatial distribution of variation of canopy CC or LAI. Blue, red and NIR bands were used for extracting texture information in variance.

One of the most common measures of image quality is the signal-to-noise ratio. Because the principal components transform (PCT) does not always produce images that show steadily decreasing image quality with increasing component number, Green et al. [34] suggested that choosing principal components is based on maximizing the signal-to-noise ratio instead of choosing new components to maximize variance, as traditional PCT does. Therefore they developed one transform method called Maximum Noise Fraction (MNF) transform to maximize the signal-to-noise ratio when choosing principal components with increasing component number. We adopted the MNF to transform ALI (9 bands), ETM+(6 bands) and Hyperion (167 bands) data to produce low-dimensional and possible predictors for mapping forest CC and LAI.

### 3.3. Prediction models

To compare capabilities of the three sensors' data for mapping forest CC and LAI, we developed six multivariate regression models with inputs of 10 selected spectral features, variables and indices (all called spectral predictors) and either CC or LAI measurements. All candidate predictors for establishing both CC and LAI prediction models with ALI, Hyperion, and ETM+ data were listed in Table 3. The 10 spectral predictors included in the prediction models were selected from all candidate variables by a stepwise regression procedure.

### 3.4. Validation

For validating forest CC and LAI results mapped with image data, we first visually interpreted CC values from a set of natural color aerial photographs with a stereoscope, then modified the CC interpreted values with actually measured CC values and calculated LAI from the modified CC values interpreted from aerial photographs. We followed the following procedure to conduct the validation.

- Step 1. Locate interpretation plots on three pseudo-color composite of ALI, ETM+ and Hyperion and the aerial photographs. Plot size was set at 2 × 2 pixels (3600 m^2^), and plots were selected based on two conditions: Being easy to locate on images/photographs and being as homogenous as possible on composite images. A total of 144 plots were selected based on the two conditions.
- Step 2. Interpret forest CC values from the 144 selected plots on aerial photographs after training for this interpretation with CC ground measured plots.
- Step 3. Modify each interpreted CC value using a relationship established between 38 ground measured CC values and the corresponding interpreted values. Then the modified CC interpreted values are used directly to verify CC results mapped with the three sensors' data in this analysis.
- Step 4. Calculate 144 LAI values from the 144 interpreted CC values in step 3 based on a relationship set up between 38 ground-measured CCs and 38 LAIs [35]. The 144 calculated LAI values (hereafter, they also be referred to as interpreted LAI values) can now be used to validate LAI maps.
- Step 5. Extract CC and LAI mapped values from the 144 corresponding plots on the CC and LAI maps whose values are predicted with the 6 prediction (multivariate regression) models.
- Step 6. Calculate root mean squared error (RMSE) and map accuracy for the 144 mapped values. Plot scatterplots of interpreted CC and LAI values against mapped CC and LAI values based on the results derived at steps 3 – 5.

## 4. Results and Analysis

### 4.1. Prediction models

Table 3 summarizes the 6 prediction models, including selected VIs, VARs and MNFs and a multiple correlation coefficient (R^2^) for each prediction model. From the table, NDVI were almost included in every model except HYP-CC that was replaced with NDWI. This indicates that the NDVI is a robustic and very useful VI to describe variations of forest CC and LAI. The spectral texture variables are also important since four modes include the variables (ALI-LAI, ETM-CC and both Hyperion models). By the table, it is apparent that all three CC relevant models had produced higher R^2^ values than the three LAI models when all models used 10 input predictors. This is because the CC value reflects real area that a sensor can “see”; it responds directly to spectral characteristics of a forest stand, and to spectra reflected from tree crowns (canopies) and underlying soil. Forest reflectance directly depends on the proportion of these different components (e.g., crown, understory and bare soil) in the elementary surface viewed by a sensor [36]. Although LAI relates to CC, they do not have a linear relationship [35]. Moreover, LAI generally reaches spectral saturation after LAI = 6 or 7 [37]. Thus, it is reasonable that the R^2^ values from the three CC related models are higher than those from the three LAI related models. Comparing the R^2^ values among the 6 prediction models, Hyperion had produced the highest R^2^ values for predicting CC and LAI, ALI was medium and the lowest was produced by ETM+ although the ALI-LAI was just slightly better than ETM-LAI.

### 4.2. CC and LAI maps

**Figure 2.**
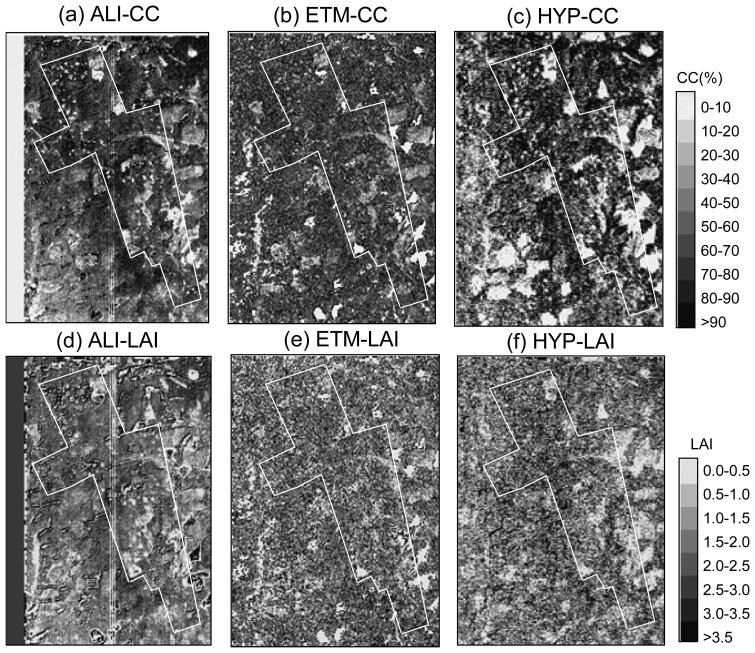
Forest CC and LAI maps produced with the three sensors' data. CC maps produced with ALI (a), ETM+ (b), and Hyperion (c) data; LAI maps produced with ALI (d), ETM+ (e), and Hyperion (f) data. The Blodgett study area is bounded in a white line in the six CC and LAI maps. In the figure, the darker the image pixels show, the higher the forest CC or LAI values.

We input pixel-based 10 predictor values to the six established prediction models (Table 3) and created six corresponding CC and LAI maps (Figure 2). Figures 2a – 2c are three CC maps (ALI-CC, ETM-CC and HYP-CC) created with 10 selected predictors, extracted from ALI, ETM+ and Hyperion data, respectively. Figures 2d – 2f are three corresponding LAI maps (ALI-LAI, ETM-LAI and HYP-LAI). The Blodgett study area is bounded by a white line in the figure. After taking a close look at these maps and comparing them with the pseudo-color composite image (Figure 1), it is evident that the three CC maps are better than the corresponding three LAI maps due to the same reason explained for Table 3; both CC and LAI maps created with Hyperion data are better than those created with either ALI or ETM+ data, especially for Hyperion data used for mapping LAI. Visually, there is a little bit of difficulty to judge which CC and LAI are better, created by ALI or ETM+. However, based on validation results, addressed as below, ALI results are better than those with ETM+ data. The three white straight lines in ALI-CC and ALI-LAI were caused by three bad detectors of ALI band 8.

**Figure 3.**
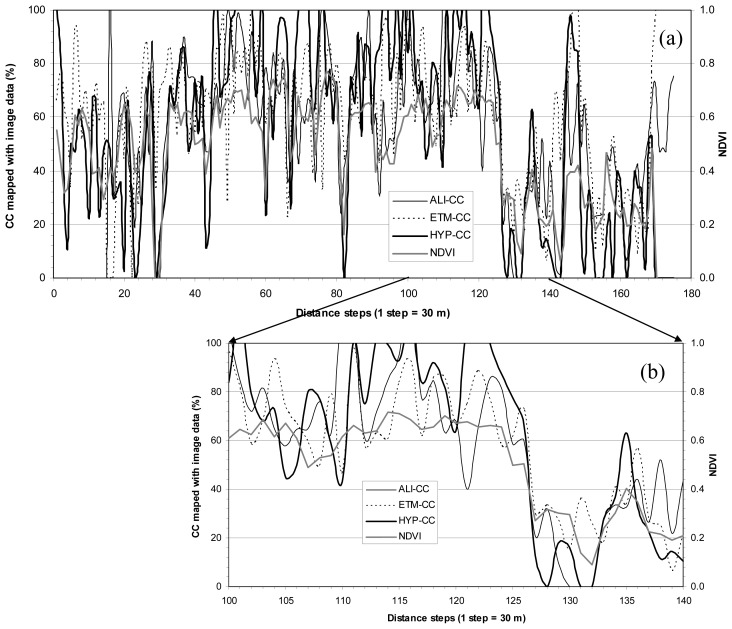

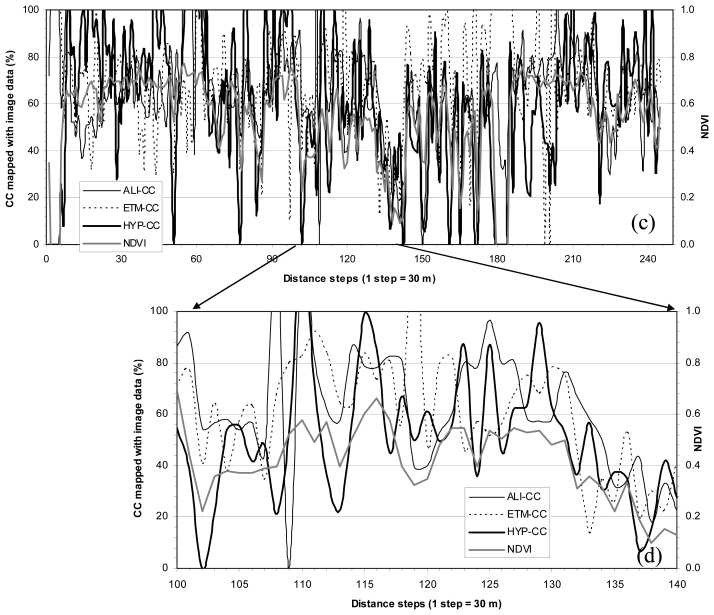
(a) CC profile (see Figure 1 for L1 location) shows variations of three CC maps: ALI-CC, ETM-CC and HYP-CC, and corresponding “Hyperion” NDVI; (b) four enlarged profiles of (a) for part of distance steps (1 step = 30 m) from 100 to 140; (c) and (d) are similar to (a) and (b) but the profile was arranged along L2 (see Figure 1).

**Figure 4.**
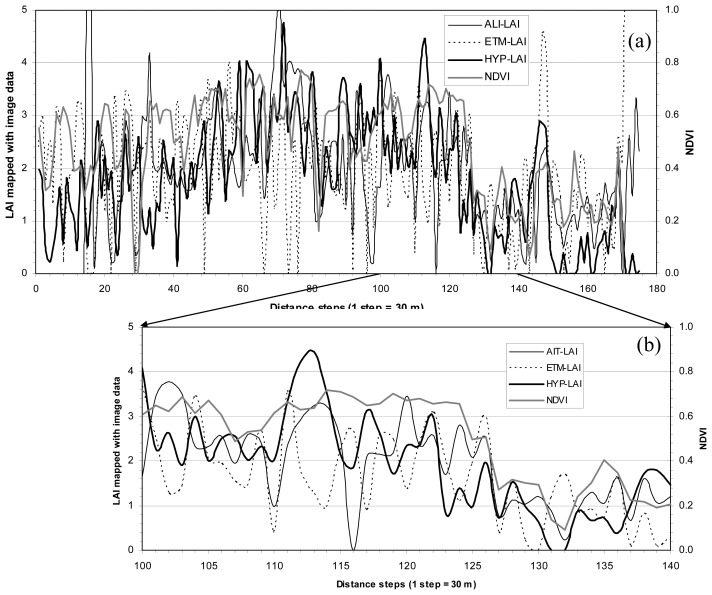

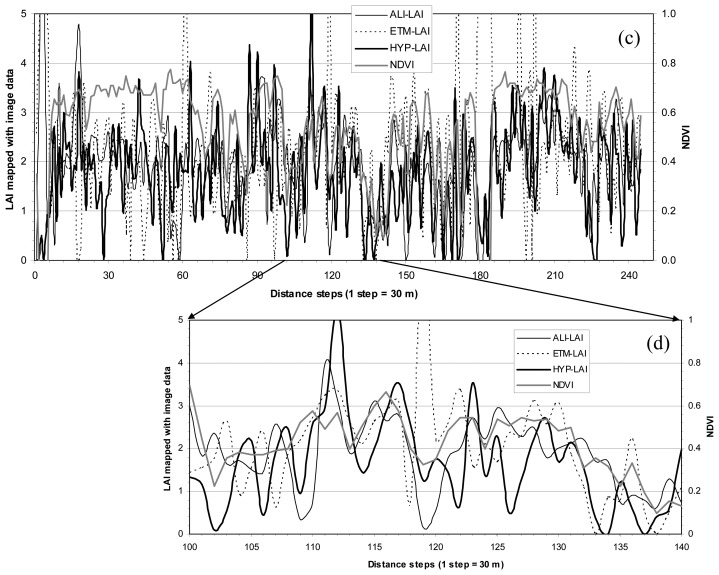
(a) LAI profile (see Figure 1 for L1 location) shows variations of three LAI maps: ALI-LAI, ETM-LAI and HYP-LAI, and corresponding “Hyperion” NDVI; (b) four enlarged profiles of (a) for part of distance steps (1 step = 30 m) from 100 to 140; (c) and (d) are similar to (a) and (b) but the profile was arranged along L2 (see Figure 1).

Figures 3 and 4 present CC and LAI profiles, respectively, along with corresponding NDVI (created using Hyperion NIR and red bands) profile along the lines L1 and L2 in Figure 1. Both figures show a generally spatial consistency among the three CC results or three LAI results mapped with the three sensors' data and NDVI. It is apparent that CC or LAI mapped with Hyperion data is more consistent with NDVI than those mapped with ALI and ETM+ data (see the enlarged profiles for CC from Figures 3b and 3d and for LAI from Figures 4b and 4d). This might be due to NDVI constructed with Hyperion data, but it could be believed that Hyperion sensor has had a greater capability to map CC and LAI than ALI and ETM+ sensors. Generally speaking, the profiles indicate that the forest CC and LAI results mapped with the three sensors' data tend consistent.

### 4.3. Validation

Due to the time difference between the three sensors' data acquisition (October 9 and 25, 2001), aerial photographs (May 25, 2000), and CC/LAI measurements (August 10-11, 2001), actual CC and LAI over the time period may have changed significantly, especially for LAI parameters. In addition, photo interpretation is dependent on the experience of the photo interpreter [38] who may make an interpretation error (usually a systematic error). In order to make the CC photo interpreted values comparable with the CC mapped values from the three sensors' data, all 144 interpreted CC values were modified according to the relationship (R^2^ = 0.923) established between CC interpreted values and ground CC measured values [35]. An exponential relationship (*LAI* = 0.595*e*^0.0174^*^·CC^*, *R*^2^ = 0.586) [35] between CC and LAI was developed from the 38 ground measured samples. The relationship was employed to calculate 144 LAI values from 144 modified CC interpreted values. Both CC and LAI values of photo interpretation were used to validate CC and LAI mapped values from the three sensors' data.

Table 4 presents some simple statistics derived from the validation results, used to judge CC and LAI map quality through comparison with the CC and LAI values derived by photo interpretation. Statistics include root mean squared error (RMSE) and mapped accuracy (MA) for each prediction model. By comparing statistical results in the table among different prediction models and between mapped results and interpreted results, it is worth noting that both mapped CC and LAI results with the lowest RMSE and highest MA values were produced by Hyperion data again, followed by ALI data, and the worst for ETM+ data.

**Figure 5.**
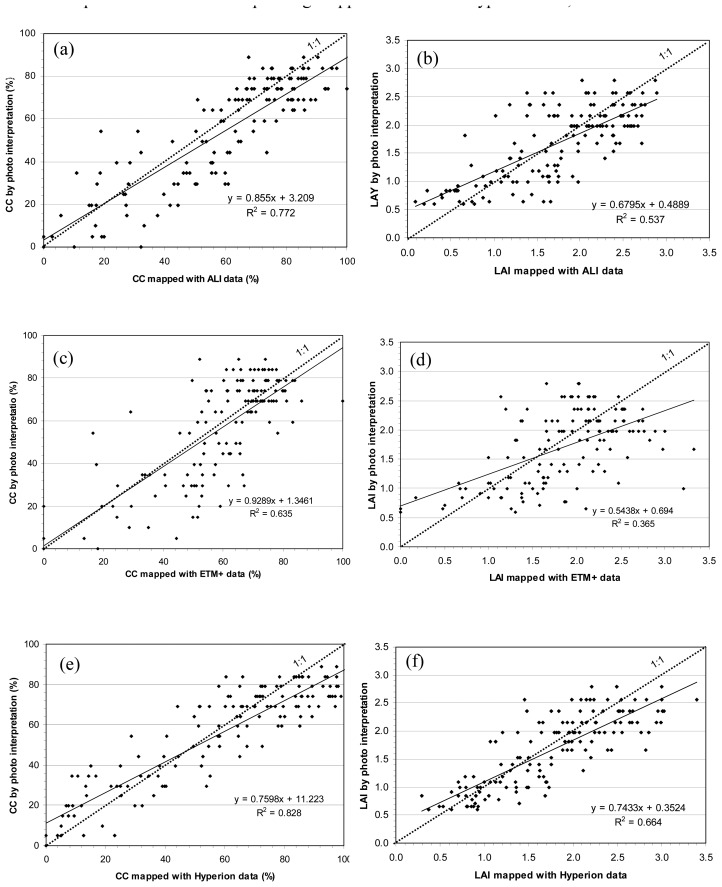
Scatter plots showing the agreement degree and reliability between the interpreted values and corresponding mapped values. (a) CC and (b) LAI interpreted values vs. corresponding mapped values with ALI data; (c) CC and (d) LAI interpreted values vs. corresponding mapped values with ETM+ data; (e) CC and (f) LAI interpreted values vs. corresponding mapped values with Hyperion data;

Figure 5 illustrates the agreement degree and reliability between mapped and interpreted CC and LAI values. The 144 CC points were plotted in Figure 5 (a, c and e) for the three CC maps produced with ALI, ETM+ and Hyperion, respectively, and the 144 LAI points were plotted in Figure 5 (b, d and f) for the three LAI maps. The more closely the scatter points distribute along the diagonal line (1:1 dash line), the better the mapped results are, thus the higher the agreement degree and reliability between the mapped and interpreted results (also the higher the correlation coefficient R^2^ of the linear relationship between mapped and interpreted values). In addition, based on the degree of closeness of a regression line (solid fine lines in the scatter plots) to its diagonal line and on how close the two lines are to being parallel, we can judge which sensor's data is best for mapping CC or LAI. Based on these criteria, it is clear that the Hyperion data again produced the better CC and LAI mapped results, again followed by the ALI data except Figure 5e that seems is not better than Figure 5c (CC mapped with the ETM+ data). However, from the distribution tendency of scattering points, it is apparent that the CC mapped with Hyperion data is much better than that with ETM+ data.

### 4.4. Performance of the three sensors for mapping CC/LAI

Based on the experimental results, it is definite that the Hyperion data outperform the other two sensors' data for mapping forest CC and LAI. This is attributed to the properties of hyperspectral data that contain rich subtle spectral information from 167 Hyperion bands. Such subtle spectral information is able to describe the slight variation of forest CC and LAI, thus it is beneficial to establish high quality prediction models. The second reason of the good performance of Hyperion data for mapping CC and LAI is the availability of those optimal VIs. The VIs extracted from Hyperion data are those constructed with these better bands (most located in SWIR and NIR regions) that have proved to have a high correlation with LAI [19]. There do exist many absorption features in SWIR and NIR, including those caused by water contents and other biochemicals [39, 40], which might relate to variations of forest canopy structure parameters, such as CC and LAI. Therefore it is highly possible that such VIs used as predictors are better than those constructed using NIR and red bands only. In fact, all VIs selected into the two prediction models with Hyperion data were constructed with those better Hyperion bands indeed except SIPI that can also be constructed with other two sensors' data. Another advantage for Hyperion sensor is its SWIR bands that are slightly affected by the atmosphere (mainly absorption) except two major water absorption bands. Therefore, the best spectral region for Hyperion is SWIR, but for ALI and ETM+, the visible region should be considered for use instead [7]. The final cause might be related to more available MNFs being selected from Hyperion data than MNFs derived from either ALI data or ETM+ data. Although the 6 MNFs and 5 MNFs selected in HYP-CC and in HYP-LAI models, respectively, do not include the first two MNFs, we believe they can be optimal combination synthesizing with other VIs and spectral texture variable. For Hyperion data, we have 20 MNFs to be selected, significantly being beneficial for establishing better prediction models than either ALI data (9 MNFs) or ETM+ data (6 MNFs), at least from the angle of mathematics.

Compared to mapping results of CC and LAI with ETM+ data, based on the validation results (Table 4, Figure 5), ALI data are better than ETM+ data although lowering the quality of LAI map (Figure 2d) produced with ALI data due to three bad detectors of ALI band 8. This is because, besides three additional bands (ALI bands 1, 6 and 7 in Table 1), ALI bands have a higher signal-to-noise ratio compared to ETM+ bands. Lencioni et al. [41] demonstrated that at 5 percent of maximum radiance, the ALI signal-to-noise ratios (SNRs) range from 100-300, while the ETM+ only manages SNRs of 15-50. The ALI push-broom system also offers greater dwelling time and significant radiometric improvement over ETM+ [2]. For the relative poor CC and LAI mapped results with ETM+ data, besides the relative low SNRs of 15-50 [41], the other possible factor is smaller number of possible predictors (13) to be selected for developing the two prediction models with ETM+ data than with other two sensors. We tried to combine with original ETM+ bands, but the mapping results were not improved.

## 5. Conclusions

In this study, a comparative analysis of performance of the three sensors for mapping forest crown closure (CC) and leaf area index (LAI) was conducted. The three sensors are Hyperspectral Imager (Hyperion) and Advanced Land Imager (ALI) onboard EO-1 satellite and Landsat Enhanced Thematic Mapper Plus (ETM+) and their data were acquired on October 9 and 25, 2001, respectively. A total of 38 mixed coniferous forest CC and 38 LAI measurements were collected on August 10-11, 2001, at Blodgett Forest Research Station, University of California at Berkeley, USA. The comparative results of the forest CC and LAI maps produced with image data and the CC and LAI measurements were used for evaluating capabilities of the three sensors for mapping forest CC and LAI.

The experimental results indicate that the Hyperion data are the most effective for mapping forest CC and LAI (CC mapped accuracy (MA) = 76.0%, LAI MA = 74.7%), followed by ALI data (CC MA = 74.5%, LAI MA = 70.7%), with ETM+ data results being least effective (CC MA = 71.1%, LAI MA = 63.4%). The analysis results prove that the Hyperion sensor outperforms the other two sensors: ALI and ETM+. This is because of its high spectral resolution that can record subtle spectral information, of its SWIR data for constructing optimal vegetation indices (VIs) that are slightly affected by the atmosphere (mainly absorption) except two major water absorption bands, and of its more available Maximum Noise Fractions (MNFs) than the other two sensors to be selected for establishing prediction models. Compared to ETM+ data for mapping forest CC and LAI, ALI data are better due to ALI data with more bands and higher signal-to-noise ratios than those of ETM+ data. From this experiment of the three sensors' comparison for mapping forest CC and LAI, the Hyperion data have demonstrated their potential of applying in forest management and ecosystem studies and the ALI sensor is proved to be a better sensor for Landsat data continuity.

## Figures and Tables

**Table 1.** Characteristics of the three sensors and a list of band numbers and wavelengths of the three sensors used in this analysis.

Parameters	EO-1/Hyperion		EO-1/ALI		Lansat-7/ETM+	

Spectral range (μm)	0.4 - 2.5		0.4 - 2.4		0.4 - 2.4	
Spatial resolution (m)	30		30		30	
Swath width (km)	7.7		37		185	
Spectral resolution	10 nm		Variable		Variable	
Spectral coverage	Continuous		Discrete		Discrete	
Number of bands	220		10		7	

Spectral bands used in this analysys	Band	WL(nm)	Band	WL(nm)	Band	WL(nm)

	1-90	430-1341	1	433-453	1	450-520
	91-124	1462-1795	2	450-515	2	530-610
	125-167	1976-2400	3	525-605	3	630-690
			4	630-690	4	780-900
			5	775-805	5	1550-1750
			6	845-895	7	2090-2350
			7	1200-1300		
			8	1550-1750		
			9	2080-2350		

Note: Band numbers of Hyperion have been re-ordered.

**Table 2.** Summary of spectral variables/indices potentially usde for establishing multivariate regression models for predicting pixel-based forest CC and LAI in this analysis

Spectral variable/index	Characteristic of the plant canopy related with the variable/index	Definition	Described by
**NDVI**, Normalized Difference Vegetation Index	Photosynthetic area; NIR region: cell structure multi-reflected spectra; SWIR region: water, cellulose, starch and lignin absorption.	(R_NIR_-R_R_)/(R_NIR_+R_R_) for ALI and ETM+; (R_1245_-R_825_)/(R_1245_+R_825_) for Hyperion;	Rouse et al. [21] Gong et al. [19]
**SR**, Simple Ratio	Same as **NDVI**	R_NIR_/R_R_ for ALI and ETM+; R_1245_/R_825_ for Hyperion;	Jordan [22] Gong et al. [19]
**NDWI**, ND Water Index	Water status	(R_860_-R_1240_)/(R_860_+R_1240_) for Hyperion and ALI.	Gao [27]
**WI**, Water Index	Water status	R_900_/R_970_ for Hyperion only.	Peñuelas et al.[26]
**LCI**, Leaf Chlorophyll Index	Chlorophyll content	(R_850_-R_710_)/(R_850_+R_680_) for Hyperion only.	Datt [29]
**PRI**, Photochemical Reflectance Index	Water stress	(R_531_-R_570_)/(R_531_+R_570_) for Hyperion only.	Thenot et al.[28]
**SIPI**, Structural Independent Pigment Index	Carotenoids: chlorophyll a ratio	(R_445_-R_800_)/(R_680_-R_800_) for all three sensors.	Peñuelas and Filella [30]
**MSR**, Modified Simple Ratio	Biophysical parameters.	(R_NIR_/R_R_-1)/((R_NIR_/R_R_)^1/2^+1) for ALI and ETM+; (R_1255_/R_824_-1)/((R_1255_/R_824_)^1/2^+1) for Hyperion.	Chen [31] Gong et al.[19]
**NLI**, Non-Linear vegetation Index	Biophysical parameters	(R^2^_NIR_-R_R_)/(R^2^_NIR_+R_R_) for ALI and ETM+; (R^2^_1200_-R_821_)/(R^2^_1200_+R_821_) for Hyperion;	Goel and Qin [32] Gong et al.[19]
**MNLI**, Modified Non-linear vegetation Index.	Biophysical parameters.	((R^2^_1760_-R_824_)*1.5)/(R^2^_1760_+R_824_+0.5) for Hyperion;	Gong et al.[19]

**Table 3.** Summary of six 10-variable regression models^a^ used for predicting pixel-based CC and LAI

Model	Band (wavelength, nm) or features included in a model	R^2^	Remarks
ALI-CC	NDVI, NDWI, MSR, NLI, MNF1, MNF3, MNF5 - MNF8	0.7712	Selected from all 17 variables: NDVI, SR, NDWI, SIPI, MSR, NLI, 2 VARs (from red and NIR bands), and 9 MNFs
ALI-LAI	NDVI, SIPI, MNF1, MNF4, MNF5 MNF7 MNF9, VAR1, VAR2	- 0.5069	Selected from all 17 variables: NDVI, SR, NDWI, SIPI, MSR, NLI, 2 VARs (from red and NIR bands), and 9 MNFs
ETM-CC	NDVI, SR, SIPI, MSR, NLI, MNF1 - MNF4, VAR1	0.6620	Selected from all 13 variables: NDVI, SR, SIPI, MSR, NLI, 2 VARs (from red and NIR bands), and 6 MNFs
ETM-LAI	NDVI, SR, SIPI, MSR, NLI, MNF1, MNF2, MNF4 - MNF6	0.5033	Selected from all 13 variables: NDVI, SR, SIPI, MSR, NLI, 2 VARs (from red and NIR bands), and 6 MNFs
HYP-CC	NDWI, WI, SIPI, MNF3 - MNF5, MNF10, MNF14, MNF20, VAR2	0.8737	Selected from all 33 variables: NDVI, SR, NDWI, WI, LCI, PRI, SIPI, MSR, NLI, MNLI, 3 VARs (from blue, red and NIR bands), and 20 MNFs
HYP-LAI	NDVI, WI, PRI, MNLI, MNF10, MNF12, MNF16, MNF17, MNF20, VAR3	0.6687	Selected from all 33 variables: NDVI, SR, NDWI, WI, LCI, PRI, SIPI, MSR, NLI, MNLI, 3 VARs (from blue, red and NIR bands), and 20 MNFs

a All six regression models simulated with 38 CC & LAI measurements.

**Table 4.** Simple accuracy statistics of CC and LAI mapped with image data against aerial photo interpretation (n = 144)

Model	RMSE^a^	Mapped accuracy (MA^b^%)
ALI-CC	13.79%	74.51
ALI-LAI	0.486	70.71
ETM-CC	15.63%	71.11
ETM-LAI	0.608	63.35
HYP-CC	13.01%	75.95
HYP-LAI	0.419	74.74

a $\text{RMSE}=\begin{array}{cc} \sqrt{\frac{1}{n}\sum_{i=1}^{n}{\left(x_{i}-{\hat{x}}_{i}\right)}^{2}} & \begin{array}{l} \text{where},\\ x_{i}\;\text{is interpreted CC or LAI value while}\\ {\hat{x}}_{i}\;\text{is corresponding CC or LAI mapped value,} \end{array} \end{array}$

## References

[1] Ungar S., Pearlman J., Mendenhall J., Reuter D. (2003). Overview of the Earth Observing One (EO-1) mission. IEEE Transactions on Geoscience and Remote Sensing.

[2] Irons J.R., Masek J.G. (2006). Requirements for a Landsat data continuity mission. Photogrammetric Engineering & Remote Sensing.

[3] Chander G., Meyer D.J., Helder D.L. (2004). Cross calibration of the Landsat-7 ETM+ and EO-1 ALI sensor. IEEE Transactions on Geoscience and Remote Sensing.

[4] Barry P.S., Mendanhall J., Jarecke P., Folkman M., Pearlman J., Markham B. (2002). EO-1 Hyperion hyperspectral aggregation and comparison with EO-1 Advanced Land Imager and Landsat 7 ETM+.

[5] Bryant R., Moran M.S., McElroy S., Holifield C., Thome K., Miura T. (2002). Data continuity of Landsat-4 TM, Landsat-5 TM, Landsat-7 ETM+, and Advanced Land Imager (ALI) sensors.

[6] Neuenschwander A.L., Crawford M.M., Ringrose S. (2005). Results from the EO-1 experiment - A comparative study of Earth Observing-1 Advanced Land Imager (ALI) and Landsat ETM+ data for land cover mapping in the Okavango Delta, Botswana. International Journal of Remote Sensing.

[7] Pu R., Yu Q., Gong P., Biging G.S. (2005). EO-1 Hyperion, ALI, and Landsat ETM+ data comparison for estimating forest crown closure and leaf area index. International Journal of Remote Sensing.

[8] Thenkabail P.S., Enclona E.A., Ashton M.S., Legg C., De Dieu M.J. (2004). Hyperion, IKONOS, ALI, and ETM plus sensors in the study of African rainforests. Remote Sensing of Environment.

[9] Goodenough D.G., Dyk A., Niemann K.O., Pearlman J.S., Chen H., Han T., Murdoch M., West C. (2003). Processing Hyperion and ALI for forest classification. IEEE Transactions on Geoscience and Remote Sensing.

[10] Lobell D.B., Asner G.P. (2003). Comparison of earth Observing-1 ALI and Landsat ETM+ for crop identification and yield prediction in Mexico. IEEE Transactions on Geoscience and Remote Sensing.

[11] Elmore A.J., Mustard J.F. (2003). Precision and accuracy of EO-1 Advanced Land Imager (ALI) data for semiarid vegetation studies. IEEE Transactions on Geoscience and Remote Sensing.

[12] Chen J., Cihlar J. (1996). Retrieving leaf area index of boreal conifer forests using Landsat TM images. Remote Sensing of Environment.

[13] White J.D., Running S.W., Nemani R., Keane R.E., Ryan K.C. (1997). Measurement and remote sensing of LAI in Rocky Mountain Montane ecosystems. Canadian Journal of Forest Research.

[14] Gower S.T., Norman J.M. (1991). Rapid estimation of leaf area index in conifer and broad-leaf plantations. Ecology.

[15] Pearlman J.S., Crawford M., Jupp D.L.B., Ungar S. (2003). Foreword to the Earth Observing 1 special issue. IEEE Transactions on Geoscience and Remote Sensing.

[16] Qu Z., Kindel B.C., Goetz A.F.H. (2003). The High Accuracy Atmospheric Correction for Hyperspectral Data (HATCH) model. IEEE Transactions on Geoscience and Remote Sensing.

[17] Goetz A.F.H., Ferri M., Kindel B., Qu Z. (2002). Atmospheric correction of Hyperion data and techniques for dynamic scene correction.

[18] Pu R., Gong P., Biging G. (2003). Simple calibration of AVIRIS data and LAI mapping of forest plantation in southern Argentina. International Journal of Remote Sensing.

[19] Gong P., Pu R., Biging G., Larrieu M.R. (2003). Estimation of forest leaf area index using vegetation indices derived from Hyperion hyperspectral data. IEEE Transactions on Geoscience and Remote Sensing.

[20] Berk A., Anderson G.P., Acharya P.K., Chetwynd J.H., Bernstein L.S., Shettle E.P., Matthew M.W., Adler-Golden S.M. (2000). MODTRAN4 User's Manual.

[21] Rouse J.W., Haas R.H., Schell J.A., Deering D.W. (1973). Monitoring vegetation systems in the Great Plains with ERTS. Proceedings, Third ERTS Symposium.

[22] Jordan C.F. (1969). Derivation of leaf area index from quality of light on the forest floor. Ecology.

[23] Turner D.P., Cohen W.B., Kennedy R.E., Fassnacht K.S., Briggs J.M. (1999). Relationships between leaf area index and Landsat TM spectral vegetation indices across three temperate zone sites. Remote Sensing of Environment.

[24] Treitz P.W., Howarth P.J. (1999). Hyperspectral remote sensing for estimating biophysical parameters of forest ecosystems. Progress in Physical Geography.

[25] Fassnacht K.S., Gower S.T., MacKenzie M.D., Nordheim E.V., Lillesand T.M. (1997). Estimating the leaf area index of north central Wisconsin forests using the Landsat thematic mapper. Remote Sensing of Environment.

[26] Peñuelas J., Piñol J., Ogaya R., Filella I. (1997). Estimation of plant water concentration by the reflectance water index WI (R900/R970). International Journal of Remote Sensing.

[27] Gao B.C. (1996). NDWI – A normalized difference water index for remote sensing of vegetation liquid water from space. Remote Sensing of Environment.

[28] Thenot F., Méthy M., Winkel T. (2002). The Photochemical reflectance index (PRI) as a water-stress index. International Journal of Remote Sensing.

[29] Datt B. (1999). A new reflectance index for remote sensing of chlorophyll content in higher plants: tests using Eucalyptus leaves. J. Plant Physiol..

[30] Peñuelas J., Filella I. (1998). Visible and near-infrared reflectance techniques for diagnosing plant physiological status. Trends in Plant Science.

[31] Chen J.M. (1996). Evaluation of vegetation indices and a modified simple ratio for boreal applications. Canadian Journal of Remote Sensing.

[32] Goel N.S., Qi W. (1994). Influences of canopy architecture on relationships between various vegetation indices and LAI and FPAR: a computer simulation. Remote Sensing Reviews.

[33] Gong P., Howarth P.J. (1992). Frequency-based contextual classification and grey-level vector reduction for land-use identification. Photogrametric Engineering and Remote Sensing.

[34] Green A.A., Berman M., Switzer P., Craig M.D. (1988). A transformation for ordering multispectral data in terms of image quality with implications for noise removal. IEEE Transactions on Geoscience and Remote Sensing.

[35] Pu R., Gong P. (2004). Wavelet transform applied to EO-1 hyperspectral data for forest LAI and crown closure mapping. Remote Sensing of Environment.

[36] Guyot G., Guyon D., Riom J. (1989). Factors affecting the spectral response of forest canopies: A review. Geocarto International.

[37] Peterson D.L., Running S.W. (1989). Chapter 10: Applications in forest science and management. Theory and Applications of Optical Remote Sensing.

[38] Biging G.S., Congalton R.G., Murphy E.C.A. Comparison of photointerpretation and ground measurements of forest structure.

[39] Elvidge C.D. (1990). Visible and near infrared reflectance characteristics of dry plant materials. International Journal of Remote Sensing.

[40] Curran P.J. (1989). Remote sensing of foliar chemistry. Remote Sensing of Environment.

[41] Lencioni D.E., Hearn D.R., Digenis C.J., Mendenhall J.A., Bicknell W.E. (2005). The EO-1 Advanced Land Imager: An overview. Lincoln Laboratory J..

